# Study on the Synthesis of ZnO Nanoparticles Using *Azadirachta indica* Extracts for the Fabrication of a Gas Sensor

**DOI:** 10.3390/molecules26247685

**Published:** 2021-12-19

**Authors:** Tirtha Raj Acharya, Pradeep Lamichhane, Rizwan Wahab, Dinesh Kumar Chaudhary, Bhanu Shrestha, Leela Pradhan Joshi, Nagendra Kumar Kaushik, Eun Ha Choi

**Affiliations:** 1 Plasma Bioscience Research Center, Applied Plasma Medicine Center, Department of Electrical and Biological Physics, Kwangwoon University, Seoul 01897, Korea; tirtharajacharya2050@gmail.com (T.R.A.); theprodip@gmail.com (P.L.); 2 Department of Physics, Saint Xavier’s College, Tribhuvan University, Maitighar, Kathmandu 44600, Nepal; 3 Chair for DNA Research, Department of Zoology, College of Science, King Saud University, Riyadh 11451, Saudi Arabia; rwahab@ksu.edu.sa; 4 Department of Physics, Amrit Campus, Tribhuvan University, Kathmandu 44600, Nepal; din.2033@gmail.com (D.K.C.); leela.pradhan@gmail.com (L.P.J.); 5 Department of Electronic Engineering, Kwangwoon University, Seoul 01897, Korea; bnu@kw.ac.kr

**Keywords:** ZnO nanoparticles, *Azadirachta indica*, functional group, thin film, ethanol sensor, sensitivity, response and recovery rates

## Abstract

This paper compared the effects of *A. indica* plant proteins over chemical methods in the morphology of zinc oxide nanoparticles (ZnO NPs) prepared by a co-precipitation method, and ethanol sensing performance of prepared thin films deposited over a fluorene-doped tin oxide (FTO) bind glass substrate using spray pyrolysis technique. The average crystallite sizes and diameters of the grain-sized cluster ZnO NPs were 25 and (701.79 ± 176.21) nm for an undoped sample and 20 and (489.99 ± 112.96) nm for *A. india* dye-doped sample. The fourier transform infrared spectroscopy (FTIR) analysis confirmed the formation of the Zn–O bond at 450 cm^−1^, and also showed the presence of plant proteins due to *A. indica* dye extracts. ZnO NPs films exhibited good response (up to 51 and 72% for without and with *A. indica* dye-doped extracts, respectively) toward ethanol vapors with quick response-recovery characteristics at a temperature of 250 °C for undoped and 225 °C for *A. indica* dye-doped ZnO thin films. The interaction of *A. indica* dye extracts helps to decrease the operating temperature and increased the response and recovery rates of the sensor, which may be due to an increase in the specific surface area, resulting in adsorption of more oxygen and hence high response results.

## 1. Introduction

Gas tracking devices are becoming increasingly popular for a variety of physical, chemical, and biological purposes. For the detection of harmful pollutant gases, flammable gases, and organic vapors, metal oxide-based chemical sensors have been widely used. Chemical sensors have several benefits, including low cost, compact size, great sensitivity, and minimal power usage [1]. Ethanol is one of the most widely used and distributed alcohols in the fields of food technology, brewing, medicine, clinical research, and biotechnology [2,3,4,5]. Excessive ethanol consumption is harmful to living creatures. In humans, ethanol vapor exposure during development can result in ventricular and septal wall thickening [2]. As a result, detecting ethanol fumes is quite important at this time. Different metal oxide semiconductor (MOSs) such as ZnO, TiO_2_, WO_3_, In_2_O_3_, MoO_3_, SnO_2_, and Fe_2_O_3_, and multi-component oxides, such as BiFeO_3_, Al_2_O_3_-ZnO, Cu-TiO_2,_ Cr-TiO_3,_ MgAl_2_O_4_, SnO_2_-Pd-Pt, SrTiO_3_, and Nb_2_O_5_-ZnO, are useful for UV sensing [6,7,8], solar cells [9], photocatalytic effects [10], and gas sensing applications [11]. Gas sensors based on the nanostructures of MOSs have a high sensitivity with short response and recovery times [12]. In addition to the chemical properties of MOSs, the surface morphology and surface-to-volume ratio also affect the relative change in resistance of MOS gas sensors [13].

Zinc oxide (ZnO) is a set II–VI *n*-type semiconductor with a vast band gap (3.3 eV) and exceptional assets, such as high exciton binding energy (60 MeV) [14], transparency in the visible region and strong infrared reflectivity [15], excellent audio characteristics and outstanding electronic chattels [16], high electron mobility (100 cm^2^ v^−1^s^−1^) [17], strong room-temperature luminescence, high chemical and thermal stability, abundance in nature, and environmental friendliness [18]. This unique property makes ZnO a proficient semiconducting material in gas sensors. The biological production of ZnO NPs utilizing plant extracts, such as leaves, roots, flowers, and seeds as a bio template, is of particular interest to researchers [12]. This green technique has various advantages, including environmentally friendliness, shorter time, lower-cost precursors, and a higher-purity result; the handling procedure is easy and does not require expensive equipment [13]. Many studies have been conducted on the green synthesis of ZnO NPs by plant leaves such as *Ixora Coccinea*, *Artocarpus gomezianus*, *Coptidis rhizoma*, *Citrus aurantifolia*, *Zingiber officinalis*, *Cyanometraramiflora*, *A. indica*, and others [19,20]. However, only a few studies on the natural synthesis of ZnO NPs using dye extracts from *A. indica* have been conducted. It is reported that the *A. indica* extracts contain approximately 140 chemical compounds [21]. The main composition of *A. indica* leaf includes crude protein (12.40–18.27%), crude fiber (11.40–23.08%), N free extract (43.32–66.60%), ether extract (2.27–6.24%), total ash (7.75–18.37%), calcium (0.89–3.96%), and phosphorus (0.10–0.30%) [22]. Incubation of benzoquinones obtained from *A. indica* leaves promoted activation of quinones, which helped to reduce particle size, according to Mathew et al. [14]. Nicole et al. reported that proteins of both high and low molecular weight play a key role in the both stability and reduction of green produced ZnO NPs [15]. Phytochemicals such as flavones, polyols, terpenoids, and plant proteins found in the *A. indica* leaf contribute functional groups of amines, alcohols, ketones, aldehydes, and carboxylic acid in bio-reduction reactions [16]. Metal compounds are converted to ZnO NPs by phytochemicals and enzymes found in the *A. indica* plant [15]. These metabolites from the *A. indica* leaf have reducing characteristics, allowing zinc ions to be quickly reduced to ZnO NPs.

The interaction of analyte gas molecules with deposited oxygen ions (O_2_^−^ or O^−^) on the MOS gas sensors determines its sensing capability, and the surface shape plays a crucial role in oxygen ion adsorption [5]. The addition of *A. indica* plant dye extracts changed the surface shape and enhanced the stability of ZnO NPs. This morphological impact in ZnO NPs is beneficial to gas sensing. A novel idea proposed in this study is the utilization of plant dye extracts in ZnO thin films for the manufacture of an ethanol gas sensor. The current research examines how *A. indica* dye extract is used to generate and analyze ZnO NPs as well as how it functions as a vapor detector.

## 2. Results and Discussion

### 2.1. Structural Analysis

**Figure 1:**
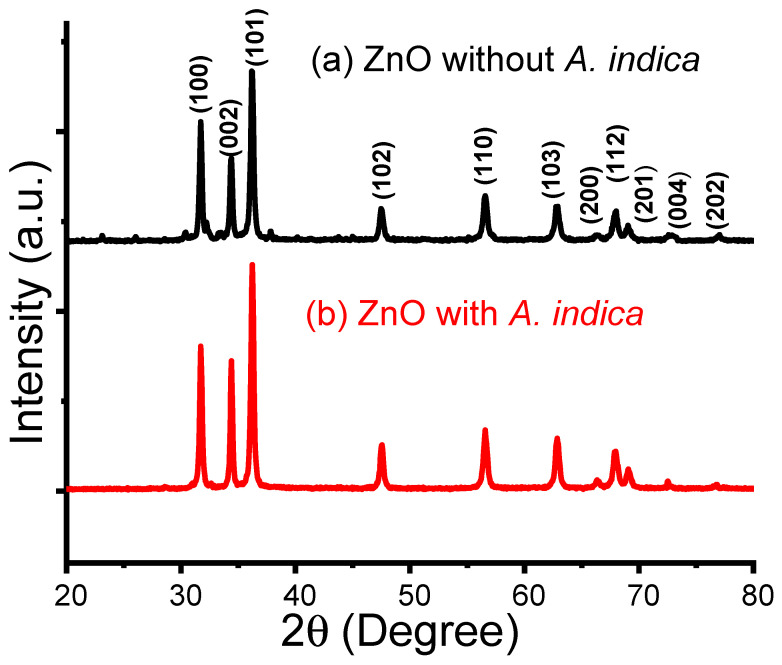
X-ray diffraction pattern of ZnO NPs prepared (**a**) without plant dye extracts and (**b**) with *A. indica* dye extracts.

The structural analysis of the prepared ZnO NPs with and without *A. indica* dye extracts was performed using X-ray diffraction (XRD). XRD testing was performed using a D_2_ Phaser (Bruker, Berlin, Germany) diffractometer with CuK_α_ radiation (wavelength = 1.54184 Å) at an operating voltage of 30 kV and a current of 10 mA. The scanning rate was 0.33 degrees per second in the 2θ range of 20° to 80°. Figure 1 shows the XRD pattern of the as-prepared ZnO NPs. The sharp diffraction peak indicates the good crystallinity of the prepared ZnO NPs [23]. Interplanar spacing (d-spacing) was obtained using Bragg’s relation: 2dsinθ = nλ [17], where λ is the X-ray wavelength, *n* is the order of reflection, and θ is Bragg’s angle. The calculated values of ‘d’ are compared with the standard JCPDS values for indexing the (hkl) planes as demonstrated in Table 1 [24]. There were slight shifts in the 2*θ* values with those of the JCPDS card number 36-1451. This may be due to the strain. The comparison of the ‘d’ values of both samples shows only a slight decrease in ‘d’ values, which may be due to the doping of the parent solution with *A. indica* dye [25].

The average crystallite size was estimated by measuring the broadening of the X-ray diffraction peaks observed in the XRD pattern using the Debye–Scherrer formula: $D=\frac{0.9\lambda }{\beta \cos\theta }$ [26], where 0.9 is the Debye constant, *λ* is the X-ray wavelength of 1.54184 Å, and *β* is the FWHM of the sharp peak. The average estimated value of the crystallite size of ZnO NPs without and with *A. indica* dye were 25 nm and 20 nm individually. The natural dye of *A. indica* leaf extracts contains different reducing factors, such as phytochemicals and enzymes, which carry out the significant reaction involved in the green synthesis of ZnO NPs. The dye extracts of leaves stabilize the NPs and stop them from aggregating [26].

### 2.2. Morphological Analysis

**Figure 2:**
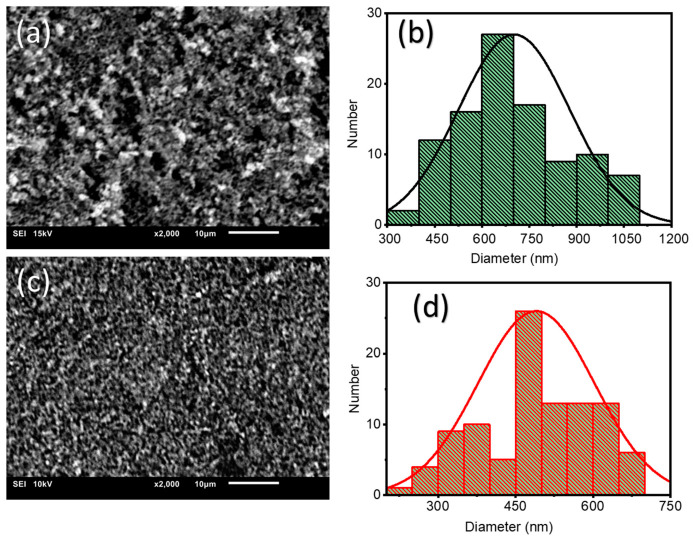
SEM images and cluster size distribution of the ZnO NP cluster at (**a**,**b**) 10 µm scale (without dye extracts) and (**c**,**d**) 10 µm scale (with *A. indica* dye extracts).

The peripheral morphology of ZnO NPs without and with *A. indica* dye extracts stood primarily using the scanning electron microscopy (SEM) (Ipvnano, Pune, India) analysis, and the subsequent images are shown in Figure 2. Using the ImageJ method [27], the diameter of the grain-sized cluster ZnO was estimated. The morphological features of both samples were considerably different based on the observed SEM estimation. The average diameter of the ZnO grain-sized NPs cluster was (701.79 ± 176.21) nm without dye extracts and (489.99 ± 112.96) nm with *A. indica* dye extracts. This finding suggests that the addition of *A. indica* dye extracts affects the morphology of thin films. Furthermore, the ZnO NPs were less aggregated and agglomerated for *the A. indica* dye extracts. This may be due to the strong kinship among them, which results in less accumulation or collection [24]. As a consequence, *A. indica* dye extracts greatly increase NP constancy and collection, and *A. indica* dye extracts lowered the diameter of ZnO NPs [25].

### 2.3. Energy-Dispersive X-ray Analysis

**Figure 3:**
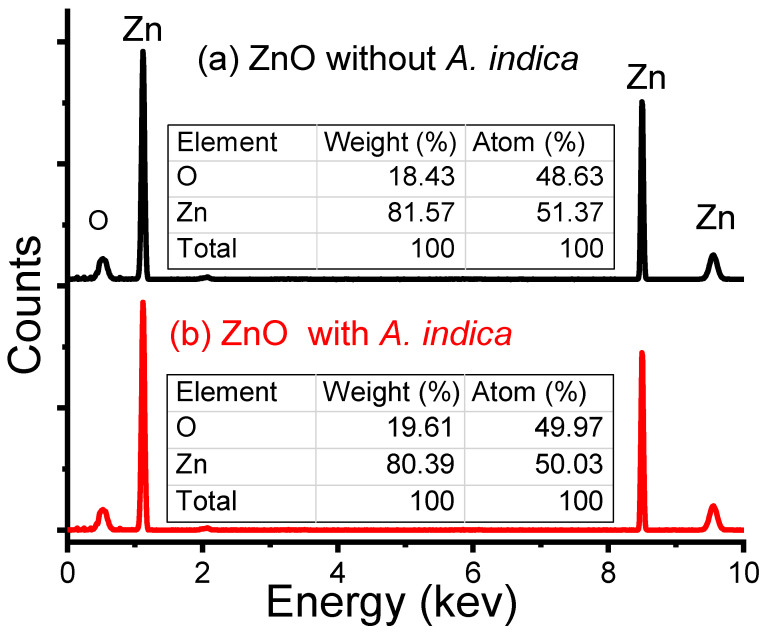
EDX spectra of ZnO NPs (**a**) without *A. indica* dye extracts and (**b**) with *A. indica* dye extracts.

Figure 3 presents the energy-dispersive X-ray (EDX) (Ipvnano, Pune, India) spectra of the ZnO NPs without and with *A. indica* dye extracts. The EDX spectra showed the presence of Zn and O in the synthesized NPs and the purity of the prepared ZnO NPs. The sharp peaks at 1.0, 8.5, and 9.5 keV in the figure indicate the appearance of zinc, and the peak at 0.5 keV reflected the presence of oxygen. The atomic composition of the bare ZnO NPs was 51.37 and 48.63% for zinc and oxygen, respectively. The atomic spreading of the *A. indica* dye extracts was 50.03% for zinc and 49.97% for oxygen. The introduction of *A. indica* dye helped to increase the oxygen ratio slightly to the prepared ZnO NPs, which may be due to the presence of water-soluble proteins from plant extracts. Thus, from the above configuration results, we can easily confirm that the synthesized ZnO NPs of both samples gained a high degree of purity. 

### 2.4. Fourier Transform Infrared Spectroscopy Analysis

**Figure 4:**
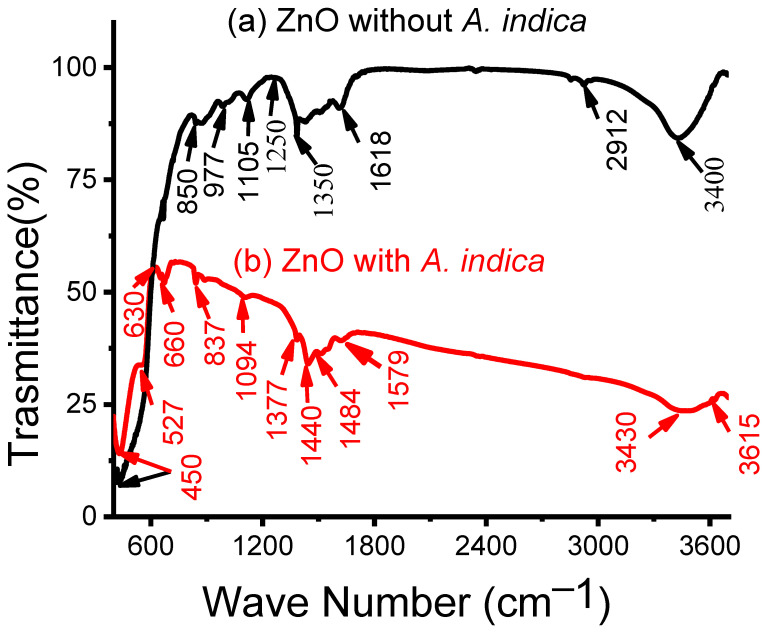
FTIR analysis of ZnO NPs (**a**) without dye extracts, (**b**) with *A. indica* dye extracts.

The Fourier transform infrared (FTIR) (Chem Tech Pro, 4100, Gujarat, India) spectrum presented in Figure 4 was applied to examine the cleanliness and arrangement of ZnO NPs without and with *A. indica* dye extracts. For both samples, the peaks at 450 and 527 cm^−1^ are the typical absorption of ZnO [28]. The spectra shown at 630–660 cm^−1^ are attributed to the occurrence of N-H stretching bonds and the peaks between 837 and 850 cm^−1^ are due to C-H stretching of aromatics [29]. The bands at 977 and 1094 cm^−1^ correspond to the C-N stretching of alcohol and phenolic groups, aliphatic amines, and aliphatic and aromatic amides [29]. Similarly, the spectrum bands illustrated between 1105 and 1579 cm^−1^ can be attributed to O-H and C-OH stretching vibration of polyols [30]. The extensive band at 1618 cm^−1^ might likely remain due to the C=C elongation of aromatic rings [30]. The band created at 2912 cm^−1^ likely correlated to the C-H stretching of polyols [31]. In addition, the bands at 3400, 3430, and 3615 cm^−1^ correlated with the O–H widening of the phenolic compounds in the dye extracts [31].

The peaks viewed in FTIR spectra of ZnO NPs after addition of *A. indica* such as 3615, 3430, 1579, 1484, 1440, 1094, 837, and 660 cm^−1^ show the presence of polyols, terpenoids, and proteins partaking functional groups of amines, alcohols, ketones, and carboxylic acid in bio-reduction responses [26]. Raphael et al. reported that proteins obtained from *A. indica* acted as a green reductant for the synthesis of NPs [32]. Terpenoids, polyols, free amino groups, carboxylic groups, alcohols, and ketones derived from *A. indica* leaf extracts play a significant role in bio-reduction reactions that are responsible for the reduction of zinc ions into ZnO NPs [23]. In addition, the several amide groups of proteins act as capping material that avoids agglomeration and makes ZnO NPs more stable [33]. Thus, the soluble substances present in the dye of *A. indica* could have acted as a capping and stabilizing agent, which averts the accumulation of NPs in the solution and plays an important part in the extracellular forge and shaping of ZnO NPs [34]. Thus, from the above results we may conclude that the soluble substances present in the dye of *A. indica* could have acted as a capping and stabilizing agent, which averts the accumulation of NPs in the solution and plays a significant role in the extracellular forge and shaping of ZnO NPs.

### 2.5. Sensitivity Analysis

**Figure 5:**
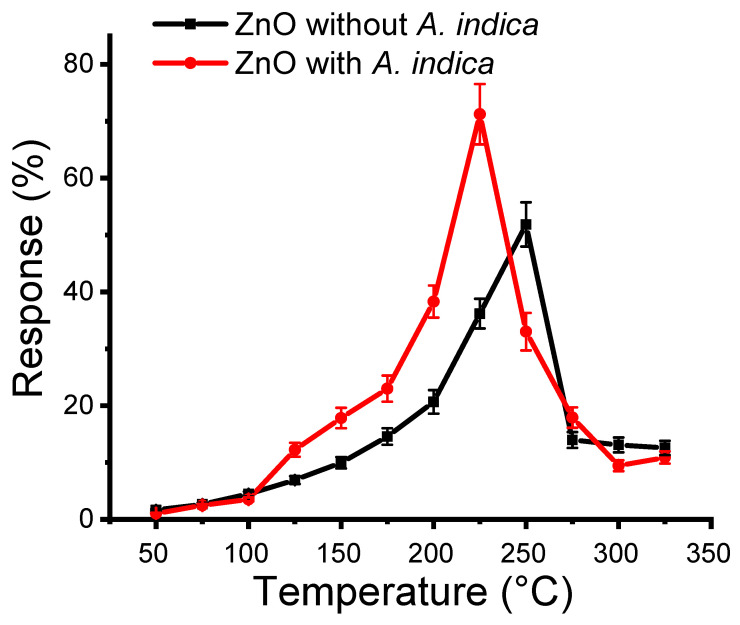
Response measurement as a function of temperature for ZnO thin films without and with *A. indica* dye extracts for ethanol vapor.

Here, thin films of ZnO without and with *A. indica* dye extracts were coated on FTO substrates. The resistance of the FTO before and after coating the ZnO thin films was measured. The resistance of the FTO increased after the coating of thin films for both samples, confirming the ZnO thin film deposition. In this study, the response of ZnO thin films was measured with respect to the change in resistance before and after the injection of ethanol vapor. The response of an N-type semiconductor of metal oxide thin film gas sensor for reducing gases can be calculated by using Equation (1) [32].

(1) $$R\;=\frac{R_{a}-R_{g}}{R_{a}}\times 100\%$$

where *R* is the response of a thin film in terms of change in resistance, *R_a_* is the air resistance, and *R_g_* is the gas resistance. One hundred parts per million of ethanol were injected into the sensing chamber, and the resistance of the ZnO thin film at different temperatures in air and ethanol vapor was measured. The sensing performance of the ZnO films was then studied. The optimization of the operational temperature is mandatory before measuring the response of the ZnO thin film. The response measurements of ZnO films without and with *A. indica* dye extract in the temperature range of 50–325 °C of ethanol vapor are shown in Figure 5. A maximum response of 51.13% and 71.95% were obtained for the ZnO thin film without and with dye extracts at an operating temperature of 250 °C and 225 °C, respectively. The introduction of *A. indica* dye extracts slightly decreased the operating temperature from 250 °C to 225 °C. The decline in operating temperature on the addition of *A. indica* dye extracts may be due to a decrease in activation energy of the reaction in the ZnO thin film [4]. Oxygen vacancies play an important role in varying the conductivity and hence the response of ZnO thin films [35,36]. On increasing the temperature, the response increases; when the thermal energy reaches the boundary to bridge the activation energy barrier of the reaction, the charge volume dramatically increases and causes a high response behavior. After crossing the optimum temperature, desorption of oxygen molecules occurs from the ZnO thin film, which decreases the response [13].

The response of an ethanol gas sensor was increased dramatically when *A. indica* dye extract was introduced in ZnO NPs. The natural capping agents present in *A. indica* dye extracts decreased both the grain and crystallite size of ZnO NPs [37]. The decrease in the grain size of ZnO NPs leads to significant increase in the specific surface area, which leads to high activity by adsorbing more oxygen molecules, and helps to enhance the response [38]. Moreover, the geometric dimensions of nanocrystalline and molecular sizes are comparable, which predetermines the difference between the kinetics of chemical transformations in nanocrystalline systems and similar processes in coarsely crystalline materials [39]. These specific features make ZnO thin films very promising for development of high-sensitivity fast-response gas sensors, in which just surface processes play the key role in the formation of a sensor signal. 

**Figure 6:**
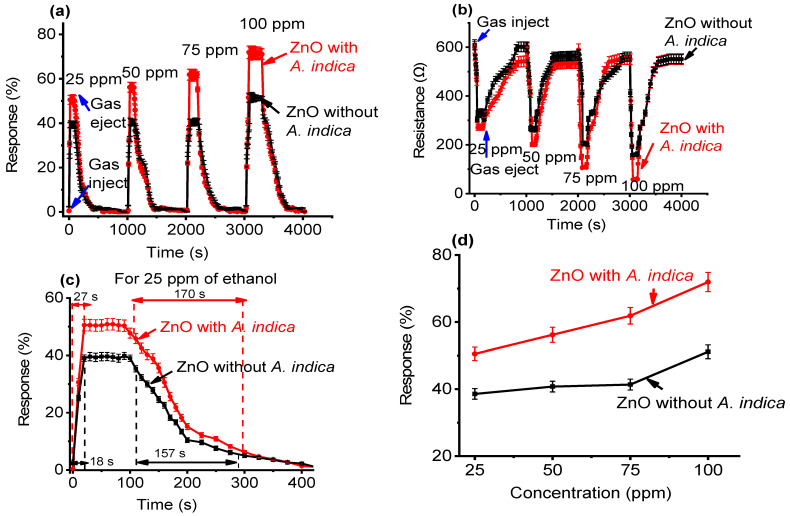
(**a**) Sensitivity measurement of ZnO thin films as a function of time. (**b**) Response curve with time scale and sensor resistance for 25, 50, 75, and 100 ppm ethanol at operating temperature. (**c**) Calculation of the response and recovery time of ZnO thin film for 25 ppm ethanol. (**d**) Sensitivity measurement of ZnO thin films as a function of concentration.

**Figure 7:**
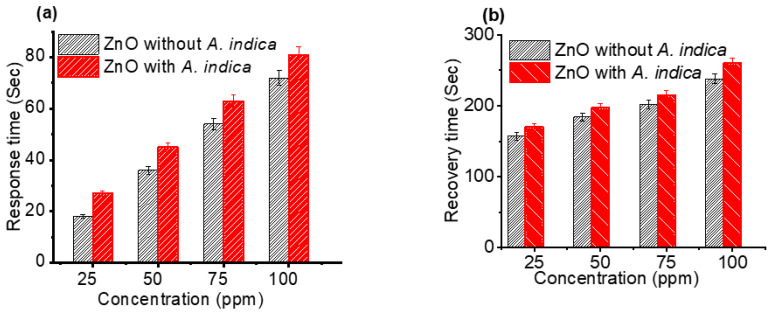
(**a**) Response time of ZnO thin films as a function of concentration and (**b**) recovery time of ZnO thin films as a function of concentration.

The response characteristics of the ZnO NPs films on the exposure of 25, 50, 75, and 100 ppm of ethanol were studied. For the purpose of computing response, each sample was analyzed five times, and the average result for each sample are displayed in Figure 6 and Figure 7. For varying concentrations of ethanol, Figure 6a shows the behavior of the ZnO thin film without and with *A. indica* dye extracts. Clearly, the response increased for higher concentration of ethanol for both cases. At 25, 50, 75, and 100 ppm ethanol, pure ZnO thin films responded with 39.58, 40.71, 40.77, and 51.13%, respectively; however, the response of *A. indica* doped ZnO thin films was 50.52, 56.19, 61.95, and 71.95%, respectively. Figure 6b depicted the curve of sensor resistance against time for ZnO thin films without and with *A. indica* dye extracts at 25, 50, 75, and 100 ppm ethanol concentrations at 250 and 225 °C. The sensor element is exposed to an air-ethanol mixture before being discharged back into the atmosphere. When exposed to air, the sensor’s resistance is strong, but it drops dramatically when exposed to ethanol. The ZnO semi-conductor gas sensor exhibited similar behavior [1,40,41]. The response rate and recovery rate are also important parameters in a gas sensor [42]. The measurement process of response and recovery times is shown in Figure 6c. For all ethanol concentrations, the response of *A. indica* dye extracts ZnO thin films is always greater than that of pure ZnO thin films, as shown in Figure 6d.

Subsequently, response and recovery times as a function of concentration were investigated, as shown in Figure 7. The average response time for bare ZnO thin films with 25, 50, 75, and 100 ppm of ethanol was 18, 36, 54, and 72 s, respectively, whereas the average response time for *A. indica* extracts was 27, 45, 63, and 81 s (Figure 7a). Similarly, the average recovery time for bar ZnO thin films with 25, 50, 75, and 100 ppm of ethanol was 157, 184, 202, and 238 s, respectively, whereas the mean recovery time for *A. indica* extracts was 170, 197, 215, and 260 s (Figure 7b). The response and recovery times of the ZnO thin film increased with the addition of *A. indica* dye extracts at ethanol concentrations ranging from 25 ppm to 100 ppm. This is due to the increased carrier concentration in the ZnO thin film after the *A. indica* dye extract doping. The oxygen adsorption process is the main factor determining the rate of response and recovery cycles [43]. The increasing response and recovery time for ZnO with *A. indica* dye extracts are attributed to the modification of the ZnO surface morphology and increased porosity between the grain- sized ZnO nanostructures, which provide more surface-active sites to promote interaction with ethanol vapor [44].

### 2.6. Sensing Mechanism

**Figure 8:**
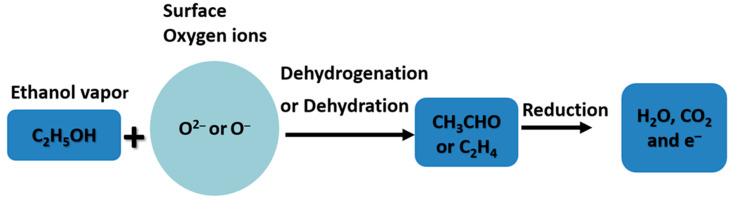
Gas response mechanism of the ZnO thin film based on ethanol vapor.

The response features of the ethanol-based gas sensor using ZnO thin films are directly linked to the modification of ZnO NPs. Here, the resistance of the thin film decreased with the supply of ethanol vapor in the sensing device, as shown in Figure 6b. The oxygen present in the atmosphere gets absorbed on the ZnO film as O^2−^ or O^−^, taking electrons from the conduction band of the ZnO and resulting in a depletion zone on the ZnO thin film [4]. This process increased the resistance of the ZnO thin film significantly. On the exposure of ethanol vapor, the ethanol vapor molecules interact with the absorbed oxygens and reinject the carrier, thereby decreasing the resistance of the ZnO thin film [45]. This effect is relatively higher in ZnO thin films using *A. indica* dye extracts than in thin films without dye. Therefore, the ZnO thin film with *A. indica* extracts shows a large gap in resistance between with ethanol vapor and without ethanol vapor in the sensing device, leading to an increase in the response of the device. The probability of the interaction of ethanol vapor with the ZnO thin film can be explained in Figure 8, as noted in previous literature [46]. 

The approved reactions are initiated by the acid–alkaline behavior of the ZnO thin film. The dehydrogenation procedure mainly occurs on the ZnO thin film with alkaline possessions; however, dehydration is preferred in the acidic region of the ZnO thin film [47]. At determined temperatures (<200 °C), more gaseous elements are adsorbed, depletion area formed on the thin film of ZnO extends intensely, providing a large possibility of interacting ethanol vapor molecules with adsorbed oxygens, thereby generating an improved response [5]. The ZnO thin film with *A. Indica* dye adsorbed more oxygen than that of the pure ZnO thin film because of large specific surface area and desorption of function groups attached with thin film at higher temperatures [48]. This effect caused high possibility of interaction of ethanol vapor molecules with oxygen, resulting in a significant decrease in the resistance of the film, which increased the response. However, at low temperatures, the surface of the ZnO thin film was not perfectly desorbed, which caused a small change in resistance. This slight change in resistance significantly decreased the sensing properties of the ZnO thin film.

## 3. Materials and Methods

### 3.1. Synthesis of ZnO NPs without Plant Extracts

ZnO NPs were prepared using the precipitation method. Initially, a 2 M zinc nitrate hexahydrate [Zn (NO_3_)_2_.6H_2_O] mixture was prepared in ethanol with continuous stirring using a magnetic stirrer for an hour (h) [49]. Concurrently, a 1 M ethanol solution of sodium hydroxide [C_2_H_7_NaO_2_] was prepared with continuous stirring using a magnetic stirrer for 2 h in a different beaker. Likewise, an ethanol solution of sodium hydroxide zinc nitrate solution was added dropwise to the zinc nitrate solution with robust stirring to produce a white precipitate. After total inclusion, the blend was allowed to settle for 1 d. Finally, the resulting precipitate was centrifuged four times at 1000 rpm (centrifuge model 800-B, China), with 10 min (m) each time, for the removal of contaminants. The precipitate was then cleaned three times using distilled water, followed by ethanol. Finally, the filtered white precipitate was dried at 120 °C and then annealed at 500 °C using a muffle furnace (BOECO Muffle Furnace, MF 8/1100, 230 V, 50/60 Hz, Lilienthal, Germany) for 12 h [49].

### 3.2. Synthesis of ZnO with A. indica Dye Extracts

**Figure 9:**
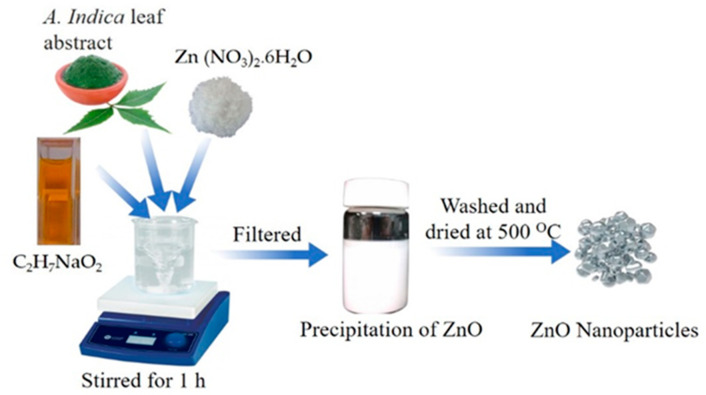
Schematics of the synthesis of ZnO using the green scheme.

Fresh leaves of *A. indica* were washed several times using distilled water and dehydrated at room temperature. The leaf extracts were then ground, 10 g of ground leaves were mixed in 40 mL of distilled water, and the mixture of leaves and distilled water was filtered to remove solid extracts. Finally, the fine solution of *A. indica* leaves and distilled water was preserved in a refrigerator at 4 °C. A 2 M zinc nitrate hexahydrate solution was prepared in distilled water under vigorous stirring. After vigorous stirring, 10 mL of aqueous leaf extracts of *A. indica* were introduced into the above solution, and a 1 M ethanol solution of sodium hydroxide was mixed in the above mixture dropwise and stirred thoroughly to obtain a white precipitate. The mixture containing the white precipitate was then stirred using a magnetic stirrer for 2 h. After stirring, the mixture was washed three times with distilled water and ethanol and then filtered. Finally, the filtered white precipitate was dried at 120 °C and then annealed at 500 °C using a muffle furnace. A schematic of the synthesis of ZnO using the green scheme is illustrated in Figure 9.

### 3.3. Synthesis of ZnO Thin Films

**Figure 10:**
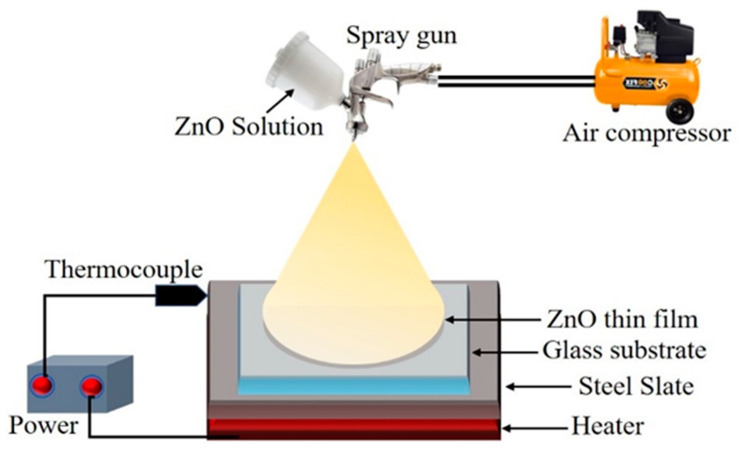
Schematic diagram of the spray pyrolysis method.

ZnO thin film was deposited on the FTO coated glass substrate using the spray pyrolysis technique. This method is especially convenient for the settling of ZnO NPs and has been a measurement method for applying a clear electrical conductor of metal oxides to a glass surface [5]. In this process, as shown in Figure 10, the spraying rate remains the same, and the back and forth motions of the machine determine the number of coats, which is digitally controlled. The main benefits of using the spray pyrolysis technique are the spherical surface shape, narrow distribution of atoms or molecules, compilation uniformity, and height controllability of the arrangement of the obtained products and their appropriateness for succeeding refining [50].

A precursor solution was prepared by taking 4.06 g of ZnO in a beaker with 50 mL of ethanol. The solution was mixed using a magnetic stirrer for 1 h at a temperature of 60 °C. The resulting solution was filtered using a filter paper and used to prepare thin films using the spray pyrolysis method. The distance from the nozzle to the glass substrate was 2.0 cm, the diameter of the nozzle was 0.5 cm, and the flow rate was maintained at 8 mL/min. The operating pressure range was maintained between 8 and 16 psi. The spray pyrolysis process included the deposition of ZnO thin films in which the dispersed solution of ZnO NPs prepared with ethanol was sprayed uniformly on the glass substrate over the heated surface at 300 °C. This method yields a very uniform thin film of ZnO owing to the constant spray rate of the nozzle of the spray gun. 

### 3.4. Sensor Application

**Figure 11:**
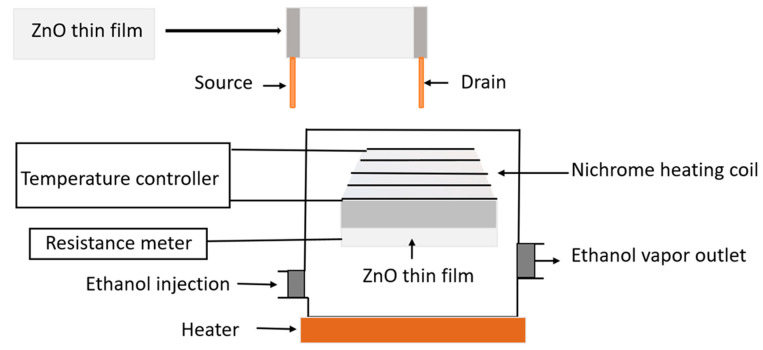
Schematic diagram of the gas sensor.

The response was measured using a custom-made ethanol gas sensor as shown in Figure 11. A nickel-chromium heater was used to heat the sensing element placed just below it, and it could be heated up to 350 °C. A thermocouple was used to monitor the temperature of the sensing element. The temperature controller was set up to maintain the temperature of the glass substrate from 90 °C to 350 °C. The source and drain were fabricated in the ZnO thin film with the help of insulating copper wire using the silver paste and then connected to a fluke multimeter (FLUKE-115, Everett, WA, USA) for resistance measurement. To measure the resistance after injecting the gas, the glass chamber was made airtight. The circuit configuration of the gas sensor was checked by using current-voltage characterization followed by ohmic behavior. The total volume of the sensor chamber is one liter (L). The distance between the base of the sensor chamber and the ZnO thin film is 5 cm. A syringe injects a fixed amount of liquid ethanol (25, 50, 75, or 100 ppm) into the airtight test chamber to achieve the desired concentration. The ethanol concentration calculation method is described in Equations (2)–(4) [51]:

(2) $$V_{\mathrm{ethanol}\;\mathrm{gas}}\;=\;C\times V_{s}$$

where *V_ethanol gas_* = volume of gaseous state ethanol, *C* (ppm) = concentration of the ethanol gas, and *V_s_* = volume of tested chamber with, *PV* = *nRT* [51].

(3) $$n_{\mathrm{ethanol}}\;=\frac{PV_{ethanol\;gas}}{RT}\;=\frac{PCV_{s}}{RT}$$

where *V* = $\frac{Mn}{\rho }$.

(4) $$V_{\mathrm{inject}}=\;\frac{Mn_{ethanol}}{\rho }=\;\frac{MPCV_{s}}{R\rho T}=\;\left(0.69\;\times \;\frac{C}{T}\right)\;\mathrm{mL}$$

The molecular weight of the liquid is *M* (g/mol), the density is *ρ* (g/mL), and the average temperature of the test chamber is *T* (K). The values of *M*, *P*, *Vs*, and *R* in our work are 46 g/mol, 101,325 Pa, 1 L, 8.31441 J/(mol K), and 0.816 g/cm^3^. Equation (4) can be used to compute the ethanol intended concentration.

## 4. Conclusions

In summary, ZnO NPs were prepared using the precipitation method. The resulting ZnO NPs were coated on glass substrates using the spray pyrolysis method to prepare the thin film. The structural, morphological, and dimensional analyses of the ZnO NPs were performed using XRD, SEM, EDX, and FTIR. The XRD pattern shows that the mean crystallite size of ZnO NPs prepared without dye extracts was 25 nm and that of the *A. indica* dye extracts was 20 nm. The SEM image shows the change in morphology of ZnO NPs from more congregated to less aggregated groups after the addition of *A. indica* dye extracts. The EDX spectra obtained to determine the percentage composition of zinc and oxygen in the synthesized samples also indicated that the prepared ZnO NPs were pure. The FTIR analysis showed the characteristic absorption band of the ZnO bond at 450 cm^−1^ and also revealed the presence of water-soluble proteins such as amines, alcohols, ketones, and carboxylic acid from *A. indica* dye extracts. The results of the sensitivity measurements with the ZnO NPs show high sensitivity toward *A. indica* dye extract thin films with a figure of 71.95% for 100 ppm of ethanol. The ZnO thin film with *A. indica* shows significantly decreased sensor resistance for 25, 50, 75, and 100 ppm of ethanol vapor compared to that of the ZnO thin film without dye extracts. The response and recovery times of the ZnO thin film were higher than those of the bare ZnO thin film between 25 ppm and 100 ppm of ethanol. The sensitivity measurement and calculation of response and recovery for 25 ppm of ethanol concentration suggest that this sensing device can also be used to detect low concentrations of ethanol. To sum up, the addition of *A. indica* dye extracts to ZnO thin films significantly increased the sensing, response, and recovery times of the ethanol-based gas sensor.

## Figures and Tables

**Table 1:** Calculation of d-spacing and crystallite size of prepared ZnO NPs.

Sample	Plane (hkl)	2θ (Degree)	FWHM (β)	d (nm) (Observed)	d (nm) (JCPDS)	Average D (nm)
ZnO NPs Without dye extracts	(100)	31.7279	0.2887	0.2817	0.2814	25 nm
	(002)	34.3859	0.2932	0.2605	0.2603	
	(101)	36.2159	0.3331	0.2478	0.2475	
	(102)	47.5432	0.4110	0.1911	0.1911	
	(110)	56.5770	0.4579	0.1625	0.1624	
	(103)	62.8518	0.5127	0.1477	0.1477	
	(200)	66.1982	0.3328	0.1410	0.1407	
	(112)	67.8183	0.4489	0.1380	0.1378	
	(201)	69.0333	0.4476	0.1359	0.1358	
	(004)	72.5773	0.3236	0.1301	0.1301	
	(202)	77.0325	0.3832	0.1236	0.1238	
**Sample**	**Plane** **(hkl)**	**2θ (degree)**	**FWHM (β)**	**d (nm)** **(Observed)**	**d (nm)** **(JCPDS)**	**Average D** **(nm)**
ZnO NPs with *A. indica* dye extracts	(100)	31.6753	0.3976	0.2823	0.2814	20 nm
	(002)	34.3385	0.2745	0.2609	0.2603	
	(101)	36.1626	0.3991	0.2482	0.2475	
	(102)	47.4574	0.3885	0.1914	0.1911	
	(110)	56.4965	0.3885	0.1627	0.1624	
	(103)	62.7772	0.4705	0.1478	0.1477	
	(200)	66.1345	0.4451	0.1411	0.1407	
	(112)	67.8529	0.5553	0.1380	0.1378	
	(201)	69.1237	0.5764	0.1357	0.1358	
	(004)	72.4312	0.6522	0.1303	0.1301	
	(202)	77.1056	0.6822	0.1235	0.1238	

## Data Availability

The data that support the findings of this study are available from the corresponding author upon reasonable request.

## Sample Availability

Not available.
